# The Effects of Graphene Stacking on the Performance of Methane Sensor: A First-Principles Study on the Adsorption, Band Gap and Doping of Graphene

**DOI:** 10.3390/s18020422

**Published:** 2018-01-31

**Authors:** Ning Yang, Daoguo Yang, Guoqi Zhang, Liangbiao Chen, Dongjing Liu, Miao Cai, Xuejun Fan

**Affiliations:** 1 The Faculty of Mechanical and Electrical Engineering, Guilin University of Electronic Technology, Guilin 541004, China; yang_ning0014@163.com (N.Y.); ldj168168@126.com (D.L.); caimiao105@gmail.com (M.C.); 2 EEMCS Faculty, Delft University of Technology, 2628 Delft, The Netherlands; g.q.zhang@tudelft.nl; 3 The Department of Mechanical Engineering, Lamar University, Beaumont, TX 77706, USA; lchen3@lamar.edu (L.C.); xfan@lamar.edu (X.F.)

**Keywords:** graphene stacking, adsorption, electronic performance, doping, first-principles theory, methane sensor

## Abstract

The effects of graphene stacking are investigated by comparing the results of methane adsorption energy, electronic performance, and the doping feasibility of five dopants (i.e., B, N, Al, Si, and P) via first-principles theory. Both zigzag and armchair graphenes are considered. It is found that the zigzag graphene with Bernal stacking has the largest adsorption energy on methane, while the armchair graphene with Order stacking is opposite. In addition, both the Order and Bernal stacked graphenes possess a positive linear relationship between adsorption energy and layer number. Furthermore, they always have larger adsorption energy in zigzag graphene. For electronic properties, the results show that the stacking effects on band gap are significant, but it does not cause big changes to band structure and density of states. In the comparison of distance, the average interlamellar spacing of the Order stacked graphene is the largest. Moreover, the adsorption effect is the result of the interactions between graphene and methane combined with the change of graphene’s structure. Lastly, the armchair graphene with Order stacking possesses the lowest formation energy in these five dopants. It could be the best choice for doping to improve the methane adsorption.

## 1. Introduction

Graphene, which possesses remarkable thermal, mechanical, and electrical properties, has become an indispensable 2D material in many electric and photonic devices [1,2,3]. For instance, graphene-based methane sensor is becoming a new research hotspot [4]. Generally, the traditional sensing materials of methane sensor are ZnO and GaAs [5,6,7], but they have many limitations because of their expensive price and hard-miniaturization. Therefore, the two-dimensional materials with a large surface area become a bright sensing material candidate in methane sensor, such as graphene, GeSe [8], antimonene [9], silicene [10,11], phosphorene [12], etc. However, GeSe, antimonene, and silicene do not have a good adsorption effect on methane. Oppositely, Refs. [13,14] have demonstrated that graphene is good to be used in a methane sensor to achieve a better performance. The good methane adsorption properties of graphene give the methane adsorption possibility of graphene heterostructures like graphene-silicene, graphene-GeSe, and graphene-phosphorene. Wu et al. have developed CH_4_ sensors using graphene nanosheets/polyaniline composite, and they have shown that the response of this sensor is up to 10 times higher than polyaniline based sensing material [13]. Yang et al. have proven that graphene has the best adsorption performance on methane compared with other gases, such as H_2_, CO, N_2_, Ar, etc. [14]. However, for now, the study of graphene on methane adsorption is still imperfect. 

Generally, graphene can be stacked in various ways to form different multilayered graphenes (MLG), which have different properties. Typical graphene stacking includes Order, Bernal (AB), and Rhombus stacking (ABC) [15,16,17]. In Order stacking, all the carbon atoms of each layer are well-aligned. For AB and ABC stacking, a cycle period is constituted by two layers and three layers of non-aligned graphene, respectively. It has been suggested that graphene’s properties like adsorption, band gap, and doping would be changed by the ways of graphene stacking [18,19,20]. Sylvain et al. found that charge carriers in few-layered graphites are highly sensitive to the layer numbers and the stacking of graphene, with ambipolar transport only existing in AB-stacked graphites [19]. Aoki and Amawashi showed that the AB-stacked MLG is semi-metal with an electrically tunable band overlap, while the ABC-stacked MLG is a semiconductor with an electrically tunable band gap [16]. The number of graphene layers has also been found critical. Amal et al. observed in experiments that the conductivity of MLG increased with the number of graphene layers after chemical doping [21]. Wang et al. found that the electrochemical interfacial capacitance of MLG sheets is dependent on the number of stacked layers [22]. Furthermore, the adsorption properties of MLG with different graphene stacking were also studied. Jonas et al. studied the adsorption of aromatic and anti-aromatic systems on graphene through π–π stacking [23]. Lu et al. investigated the influence of AB stacking on the optical properties of MLG in an electric field [24]. Based on the above discoveries, there is no doubt that these stacking ways would cause some impacts on graphene-based methane sensor. Therefore, the design and evaluation of the graphene-based methane sensor, from the aspect of stacking ways are essential and meaningful.

While the effects of graphene stacking on MLG’s properties had been well acknowledged, a comparative study of different ways of stacking is still lacking. Although the stacking effects on the adsorption, the band gap, and the doping of MLG are considered important, they are not yet understood. For instance, some research has shown that the stacking possessed a nonnegligible influence on electrical performance, but the changes are really diverse and messy. Furthermore, the studies of graphene stacking effects on methane adsorption are still lacking. In addition, doping is good for methane adsorption, such as the dopants of B, N, Al, Si, and P. The doping of Al and P would lead to a dramatic increase in methane adsorption energy, but some research revealed that the doping of Al and P in graphene is difficult. However, there are no reports on the graphene stacking effects on doping. Adsorption energy is an important and direct criterion to evaluate the adsorption effect. Band gap can partly reflect the electrical properties of graphene. In addition, doping is often used as a means to improve the performance of the target material. However, the studies of graphene stacking in these aspects are still lacking, which is exactly what we need to know. This is why we investigate the effects of graphene stacking on graphene’s properties, methane adsorption, and doping.

In this paper, the effects of graphene stacking on methane adsorption and doping are first reported using the first-principles theory. The DFT-D has demonstrated that it is more appropriate for the multilayered graphene calculations than DFT. Both the zigzag and armchair graphenes are considered. First, the effect of graphene stacking on the adsorption is studied. It is found that the AB-stacked graphene has the highest adsorption energy on methane, while the adsorption effect of the ABC-stacked graphene is the worst. Second, the changes of band gap related to different number layers are compared. The effect of stacking on band gap is significant, but it does not cause big changes to band structure and density of states. Then, the distances of methane–graphene systems are also analyzed. In the comparison of distances, the average interlamellar spacing of the Order stacked graphene is the largest. Moreover, the adsorption effect is the result of the interactions between graphene and methane combined with the change of graphene’s structure. Third, the effect of graphene stacking on the doping is discussed. The formation energies of the five dopants of B, N, Al, Si, and P in different stacked MLG are reported. The armchair graphene with Order stacking possesses the lowest formation energy in these five dopants, which is the best choice for doping to improve the methane adsorption. 

## 2. Theory and Simulations

All the calculations were performed in Dmol^3^ code base on density functional theory (DFT) [25], which is a kind of quantum mechanics simulation for the electronic structure of the multi-electron system. DFT has been widely used in the study of physical and chemical properties of nano-materials, including graphene and carbon nanotubes (CNTs) [26,27]. DFT can also accurately simulate with tens to hundreds of atomic systems and describe the atom as quantum particles, namely, the set of nuclei and electrons [28]. The Schrodinger equation is the key of DFT, which is described in Equations (1) and (2) as follows:

*H*Φ[{*R*_I_, *r*}] = *E*_tot_Φ[{*R*_I_, *r*}], (1)

*H* = *P*_I_^2^/2*M*_I_ + *Z*_I_*Z*_J_ e^2^/*R*_IJ_ + *p*^2^/2*m*_e_ + e^2^/r − *Z*_I_ e^2^/|*R*_I_ − *r*|, (2)

where *R*_I_ and *r* are the coordinates of the atomic nucleus and the electron, respectively. Equations (1) and (2) describe a time-independent and solvable Schrödinger equation, where the first two terms of Equation (2) represent the kinetic energy of atoms and electrons, respectively, and the latter three items denote the system Coulomb exclusion potential. The generalized gradient approximation (GGA) and local density approximation (LDA) are the exchange–correlation functionals commonly used in quantum mechanics calculation. GGA was chosen in this theoretical study and can be described in Equation (3):

(3) $$E_{xc}\left[\rho \right]=\int f_{\mathrm{xc}}[\rho \left(r\right),\;\left|\Delta \rho \left(r\right)\right|dr],$$

where the exchange–correlation energy of inhomogeneous electron gas is replaced by the $E_{xc}\left[\rho \right]$ of uniform electron gas. Furthermore, in this calculation, the ultrasoft pseudo potential is used to describe the interaction between electrons and ions. The cutoff energy is at 280 eV, the Brillouin zone is sampled using a 9 × 9 × 1 Monkhorst–Pack [29] k-point grid, and Methfessel–Paxton [30] smearing is at 0.005 Ha. The convergence criterion of self-consistent field energy is 1.0 × 10^−6^ eV, and the MAX force is 0.002 Ha/Å.

## 3. Results and Discussion

Because of the sheet structure of graphene, the multilayered graphene should be restricted by Van der Waals force. However, DFT calculations cannot estimate the weak interaction, which would lead to the poor calculation accuracy of methane adsorption. In order to solve this problem, the dispersion correction in DFT (DFT-D) was introduced in our work [31].

In the semiempirical dispersion-correction approach, the missing dispersion contribution to the interatomic interaction is approximated by a simple isotropic potential. At long range, this potential is given by the *C*_6,ij_*R*_ij_^−6^ term, where *C*_6,ij_ is a material-specific, so-called dispersion coefficient between any atom pair *i* and *j* at distance *R*_ij_. At short range, the long-range expression is matched to the DFT potential by multiplication with a damping function f (*R*_ij_°, *R*_ij_), which reduces the additional dispersion contribution to zero, subject to a cutoff defined by some suitably calculated combination *R*_ij_° of the vdW radii of the atom pair. The dispersion-corrected exchange-correlation functional is then formed by simply adding the correction potential to the ordinary DFT exchange-correlation functional. As *C*_6,ij_ coefficients are additive, the dispersion-corrected total energy *E*_tot_ can be written as:

(4) $$E_{\mathrm{tot}}=E_{\mathrm{DFT}}+s_{i}\sum_{i=1}^{N}\;\sum_{j>i}^{n}f\left(S_{R}\;R_{\mathrm{ij}}{}^{\circ},R_{\mathrm{ij}}\right)C_{6,\mathrm{ij}}{R_{\mathrm{ij}}}^{-6},$$

where *E*_DFT_ is the standard DFT total energy and the sums go over all N atoms in the system.

Much research has demonstrated that DFT-D can improve the calculation accuracy. Dappe et al. found that the interlayer equilibrium distance of graphene calculated by DFT-D is more closely with the experimental evidence [32]. Vanin et al. used it to study the properties of graphene on metals [33]. Zheng et al. showed that the PBE0-TS and PBE-Grimme functionals are able to describe the Van der Waals interaction of a solid methane well, and the performance of PBE-TS is also acceptable [34]. In addition, Le et al. gave a comparative Van der Waals study of nucleobases physisorption on graphene [35]. All of the above studies have shown that the DFT-D calculation for graphene is feasible. In this study, the zigzag graphene with Order stacking is taken as an example to compare the methane adsorption effects calculated by DFT and DFT-D, respectively. 

Generally speaking, adsorption energy can be a criterion to evaluate the adsorption effect, namely, the value of adsorption energy is bigger, and the adsorption effect is better. The adsorption energy (*E*_a_) is defined in Equation (5):

*E*_a_ = *E*_graphene + gas molecule_ − (*E*_graphene_ + *E*_gas molecule_), (5)

where *E*_graphene + gas molecule_ is the total energy of graphene adsorbed system, *E*_graphene_ is the energy of pristine graphene, and *E*_gas molecule_ is the energy of the gas molecule. 

The adsorption energy and the distances including the average interlamellar spacing of optimized graphene (d1) and optimized methane-graphene (d2) and the distance between graphene and methane of optimized methane-graphene (d3) are all calculated by DFT and DFT-D (PBE-grimme) and are listed in Table 1. Based on the adsorption energies and the distance, the methane adsorption on graphene is the physical adsorption that the molecular force only exists between adsorbate and adsorbent. It is obvious that the adsorption energies in DFT calculation are smaller than that of DFT-D. In addition, the distances in DFT calculation are very big, whereas the distances in the DFT-D calculation are approximately 3.4 Å, which agrees with the previous studies [36]. This demonstrated that the DFT-D method is more appropriate for the graphene calculations. Consequently, the DFT-D with the exchange-correlation of PBE-grimme is used in the whole calculations.

**Figure 1:**
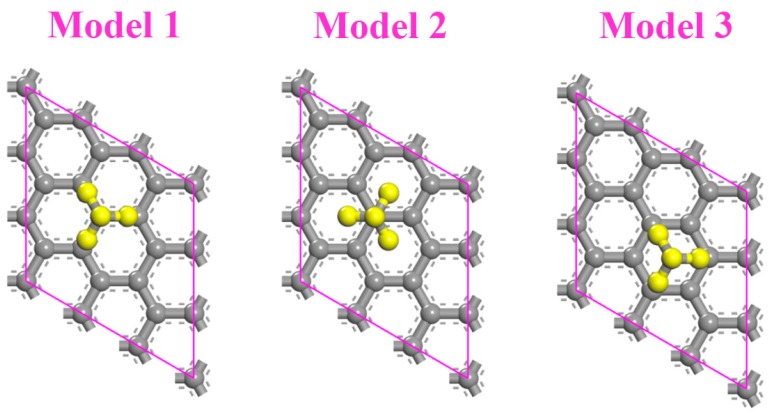
Three different methane adsorption models in monolayer graphene.

It is well known that the adsorption site has some important impacts on adsorption effects. Therefore, different adsorption models were firstly discussed in our calculations. In Figure 1, three different methane adsorption models are presented. Both the C and H atoms of methane are on the top of the C atoms of graphene in model 1, while the H atoms are on the top of the carbon ring center in model 2. In model 3, the C atom of methane is on the top of the carbon ring center, and the H atoms are on the top of the C atoms. The adsorption energy, the distance between methane and graphene, and the band gap of these three models are listed in Table 2. Compared with zigzag graphene, the adsorption energy of the armchair graphene is a little smaller. However, the electronic performance of the armchair graphene is better, for which all of the band gaps are 0. It is noteworthy that the adsorption energy of model 2 is the largest in both zigzag and armchair graphene. Furthermore, the distance of model 2 is the least. This means that the adsorption effect is better. We suggest that the H atoms in methane do not directly face the C atoms of graphene, which reduces their repulsive interaction. On the other hand, as in the multilayered graphene, the methane is always put on the first layer surface. The model that possesses the highest adsorption energy in monolayer graphene was chosen as the adsorption model in the stacked graphene to study the effect of stacking on methane adsorption. Based on the above analysis, model 2 is selected in the following studies.

### 3.1. The Effects of Stacking on Adsorption

**Figure 2:**
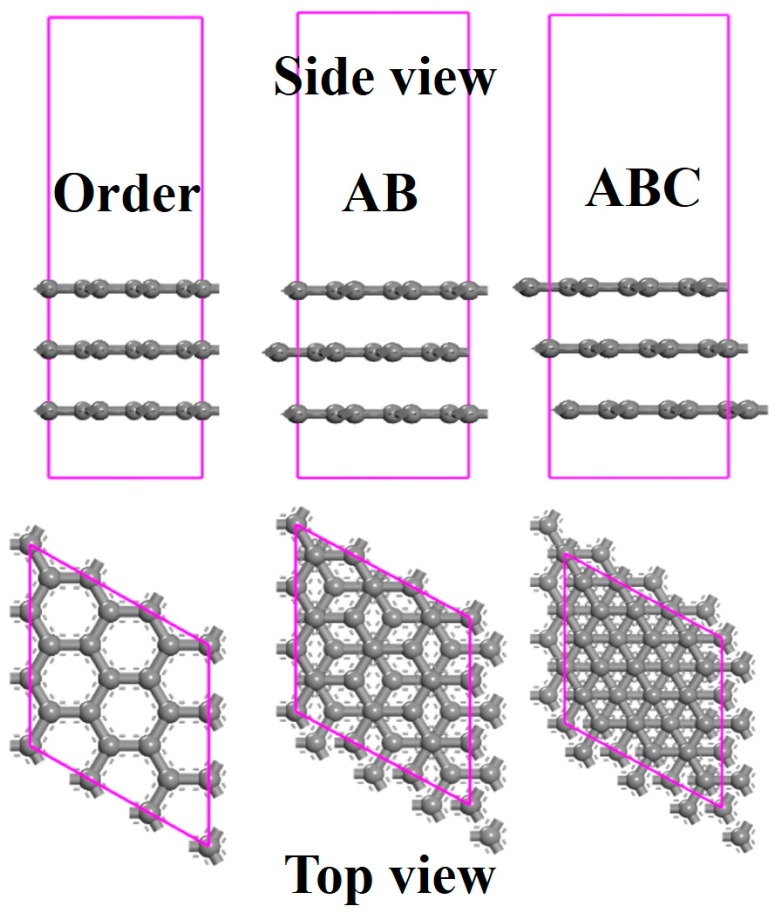
The side and top views of the stacked graphenes with Order, AB, and ABC pattern.

Taking an example of zigzag graphene, Figure 2 shows the structures of the three layers of graphenes with Order, AB, and ABC stacking, respectively. The interlamellar spacing of graphene is set as 2.65 Å, and the distance between methane and graphene is set as 2.5 Å. In Order stacking, all the carbon atoms of each layer are well-aligned. For AB and ABC stacking, a cycle period is constituted by two layers and three layers of non-aligned graphene, respectively. A highly symmetrical structure occurs in Order stacking, while the structure in ABC stacking is relatively complicated. 

**Figure 3:**
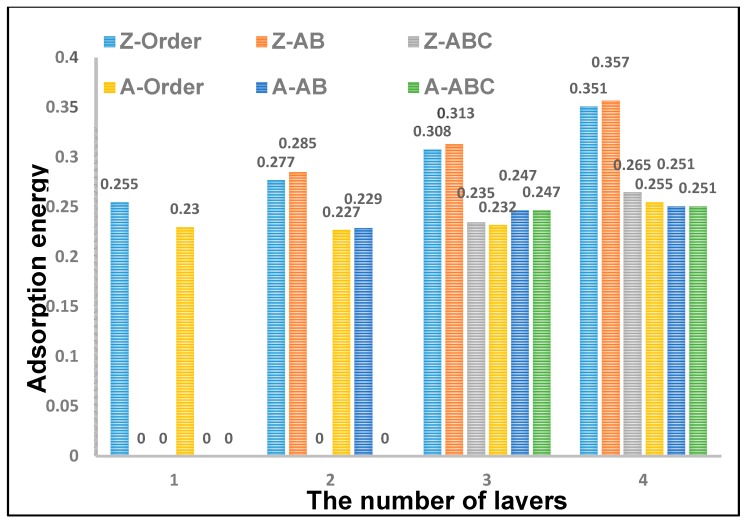
The comparison of adsorption energy between six types of the stacked graphenes with one to four layers; the Order, AB, and ABC stackings of zigzag- and armchair-graphenes are represented by Z-Order, Z-AB, Z-ABC, A-Order, A-AB, and A-ABC, respectively.

Graphene has a large surface area and porosity. As a result, it has excellent adsorption properties of gases and incomparable advantages in the production of gas sensors. Graphene has a better adsorption effect on methane compared with other gases. We know that the natural graphene often has many stacked ways, such as Order, AB, and ABC, so what are the effects of stacking on graphene-based methane sensor? A comprehensive research of stacking effects on methane adsorption of graphene is learned in this section. Model 2 is selected as the reference standard. Figure 3 shows the comparison chart of the adsorption energy in differently stacked graphene with one to four layers. Here, the Order, AB, and ABC stackings of zigzag- and armchair-graphenes are represented by Z-Order, Z-AB, Z-ABC, A-Order, A-AB, and A-ABC, respectively. From the picture, it is obvious that the adsorption energy of Z-AB is the biggest; the maximum value is 0.357 eV. The second biggest value belongs to Z-Order; its maximum value is 0.351 eV. The values of 0.357 eV and 0.351 eV are very close, which can be considered that the adsorption energies are equal in DFT calculations. The adsorption energies of other stacked graphenes are a little smaller; the value ranges from 0.23 eV to 0.27 eV. Generally, the adsorption effect of zigzag graphene is better than that of armchair graphene. The monolayer graphene does not involve stacking. Thus, it is just divided into zigzag and armchair types. Because ABC stacking is a three-layered structure, only Order and AB stacking are compared in the two-layered graphene. The adsorption energy of AB stacking is bigger than that of Order stacking. This situation is the same as in the three- and four-layer graphenes. Overall, the adsorption energy of zigzag graphene is bigger than that of armchair graphene. AB stacking has the highest adsorption effect among these three stacked ways. In addition, it also should be noted that all the adsorption energy increases with the number of the graphene layer.

### 3.2. The Effects of Stacking on Electronic Properties, Such as Band Gap and Density of States

**Figure 4:**
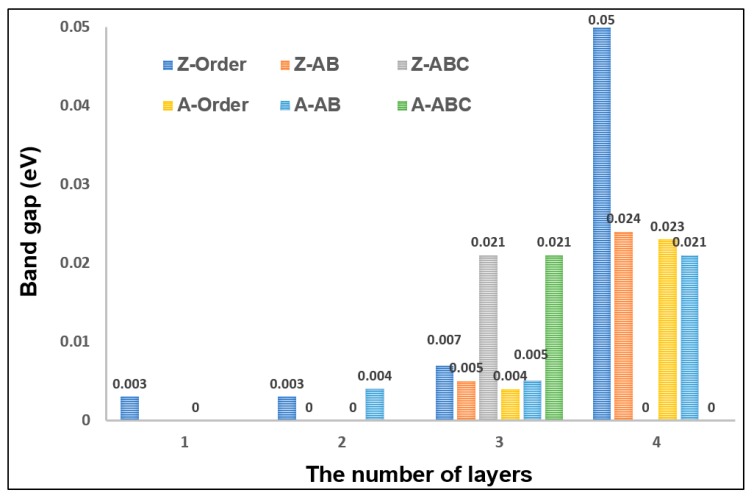
The comparison of the band gap of six types of the stacked graphene with one to four layers, such as Z-Order, Z-AB, Z-ABC, A-Order, A-AB, and A-ABC.

**Figure 5:**
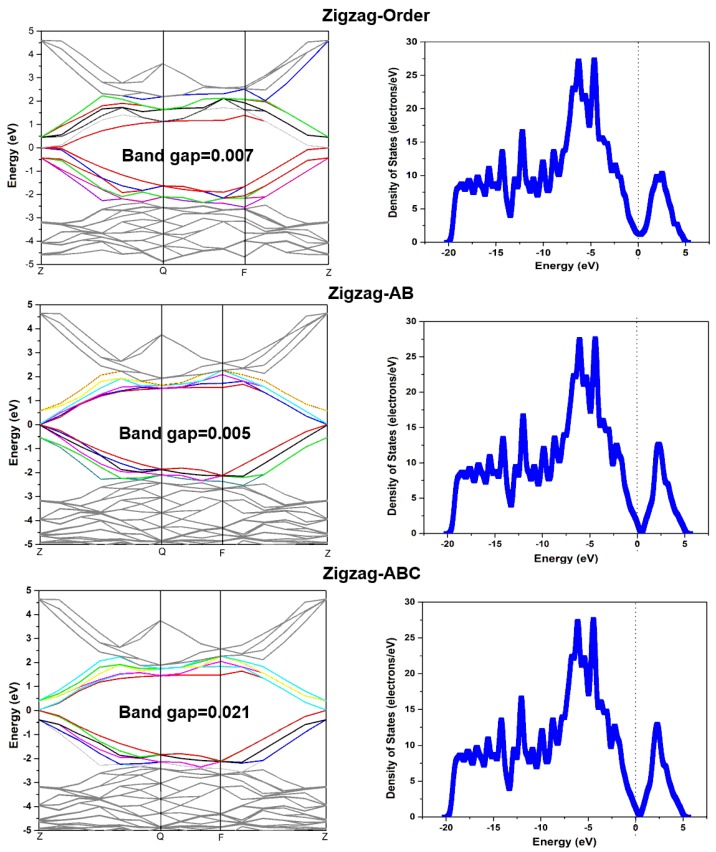
The schematic diagrams of the band structure and the density of states in the three layers of zigzag graphene with Order, AB, and ABC stacking, respectively.

Graphene adsorption is closely related to the change of electronic properties. In this section, the electronic properties of the graphene with different stacking are analyzed. As shown in Figure 4, the changes of the band gap of the differently stacked graphenes are compared. In general, the three-layered graphene with ABC stacking and four-layered graphene with Order stacking process a larger band gap. Oppositely, the AB-stacked graphene has a smaller band gap. At the same time, the AB-stacked graphene also has a better adsorption effect on methane. This demonstrates that the AB-stacked graphene is a good choice for methane adsorption. As for the ABC-stacked graphene, the band gaps are the same as in the three-layered zigzag and armchair graphenes, and the value is up to 0.021 eV, whereas, they suddenly decrease to zero in four-layered graphene. We suggest that this is because of the periodic structure of the three-layered graphene. In addition, the band gap has an abrupt uprush in four-layered graphene with Order stacking. This is caused by the multilayered structure of graphene in which the interlamellar distance has a big change. In order to more fully analyze the influence of the stacking on the electronic characteristics of graphene, the band structures and density of states (DOSs) are compared, as shown in Figure 5. Owing to the band gap values of the armchair and zigzag graphene with three layers are very similar, we just choose the Z-Order, Z-AB, and Z-ABC graphenes to illustrate their electric properties. There is not much difference in these three band structures, but the band structures of AB and ABC are more similar. Like the band structure, the situation happens in the DOSs diagram, too; however, the difference is that the Fermi level is not located in the zero of the Z-Order graphene. Consequently, we can draw a conclusion that the stacking does not cause big changes to the band structure and DOSs of graphene; it only has some changes in the band gap.

### 3.3. The Effects of Stacking on the Distance of Average Interlamellar and the Distance between Graphene and Methane

To understand the mechanism of stacking effect on methane adsorption, some distances in six types of stacked graphene are recorded in Table 3, including the average interlamellar spacing of optimized graphene (d1) and optimized methane-graphene (d2) and the distance between graphene and methane of optimized methane-graphene (d3). Generally speaking, by comparing all the distances, d1 decreases with the increase of the layer number. Furthermore, after methane is adsorbed, d2 will be further reduced, but the change is very small. However, d3 is opposite to d1 and d2; it will be increased with the layer number. Comparing with the dates in detail, we could find that the distances (d1, d2, and d3) of armchair graphene are bigger than that of zigzag graphene. This is why the adsorption energy of zigzag graphene is bigger than that of armchair graphene. In addition, both d1 and d2 of the Order stacked graphene are the largest among these three types of stacked graphenes. The maximum values are 3.450 Å and 3.454 Å in the A-Order graphene, respectively. The distances of d1 and d2 in the AB-stacked graphene are the smallest. The minimum value is 3.282 Å in the four-layered Z-AB graphene. The distances of d1 and d2 in the ABC-stacked graphene are close to the AB-stacked graphene. Beyond these, there are some important things that we must pay attention to. We note that d3 increases with the layer number; similarly, the adsorption has this variation tendency, too. This is contrary to the rules that the distance is smaller, and the adsorption energy is bigger. In addition, the d3 of the AB-stacked graphene is also not the smallest. We suggest that the adsorption effect is the result of the interactions between graphene and methane combined with the change of graphene’s structure. The decrease of d2 can strengthen the methane adsorption effect. Therefore, although d3 becomes a little bigger with the increase of layer number, the methane adsorption is strengthened.

### 3.4. The Effects of Stacking on Doping

**Figure 6:**
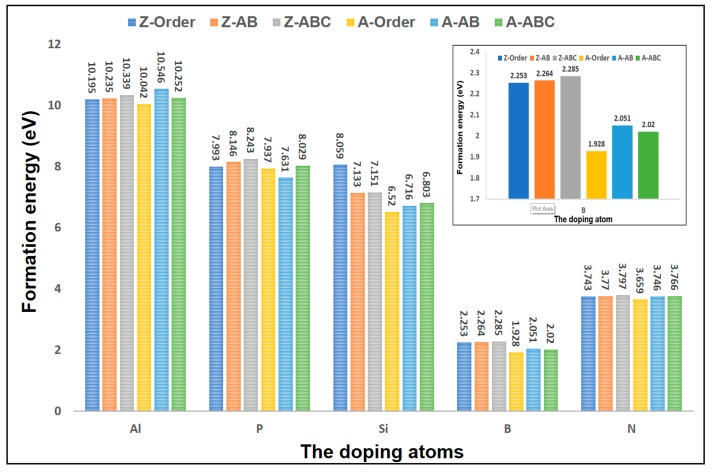
The formation energy of the stacked graphene with three layers doped with five atoms, namely, Al, P, Si, B, and N. The inset illustrates the formation energy of the stacked graphene doped with B.

Previous studies have found that the doping in graphene is helpful to the adsorption of gases [37,38], such as the atoms of B, N, Al, Si, and P. According to our calculation, these dopants did improve the methane adsorption, especially the doping of Al and P. This makes the adsorption energy increase sharply to 2.5 eV and 2 eV, respectively. The doping of B, N, and Si makes methane adsorption reach 0.55 eV, 043 eV, and 1 eV, respectively [14,39]. Obviously, doping has a positive effect on methane adsorption. Consequently, the study of stacking effects on doping is also important. In this section, we will conduct specific research on the stacking effect on doping. The formation energy can be used as the judgment of whether the feasibility of the atoms for doping is good or not. The smaller the formation energy is, the easier the doping becomes. The formation energy [40] is described in Equation (6):

*E*_formation_ = *E*_(grapheme +__d)_ + *nE*_C_ − *E*_graphene_ − *nE*_d_, (6)

where *E*_formation_ is the formation energy, *E*_graphene_ is the energy of pristine graphene, and *E*_C_ and *E*_d_ are the chemical potentials determining carbon and doping atoms. The three-layered graphene and the atoms of B, N, Al, Si, and P are chosen to calculate their formation energy, as shown in Figure 6. First, the formation energy in the armchair doped-graphene is smaller than that of the zigzag doped-graphene, which means that the doping of these atoms is easier to implement in armchair graphene. Second, the formation energy of Al, P, and Si is larger, which is very detrimental to doping; their values are about 10 eV, 8 eV, and 7 eV, respectively. Although these dopants can make methane adsorption have a great improvement, the cost is very high. Instead, the formation energy of B and N is much smaller, which will be good for the doping. In addition, the minimum value of formation energy is 1.928 eV, which occurred in the A-Order graphene doped with B. In addition to this, the doping of B and N is also obviously helpful to the methane adsorption. Last, we should note that the A-Order stacking processes the smallest formation energy in all types of stacked graphene. The A-Order is the best graphene structure for doping. The inset presents the formation energy of the stacked graphene doped with B. It can be seen that the gap of the formation energy among them is large; there is a 16% difference between the maximum and minimum value of formation energy. Therefore, based on the consideration of doping and adsorption, A-Order stacking is the best choice for methane adsorption. Of course, the A-AB stacking is good, too.

Obviously, the graphene stacking methods have great influence on the properties of graphene, methane adsorption, and doping. As for methane adsorption and doping, AB stacked zigzag graphene with four layers has the best adsorption capacity, while Order stacked armchair graphene has the better doping performance. In addition, the armchair graphene possesses better electrical properties, but it has larger layer spacing than that of zigzag graphene. Therefore, the type of graphene should be selected according to the purposes and requirements of the sensing material.

## 4. Conclusions

In this paper, the effects of Order, AB, and ABC stacking on the properties of graphene were investigated comprehensively via first-principles theory. Comparing the adsorption sites of methane, model 2 is the best choice for methane adsorption. The adsorption energy of the Z-AB graphene is the largest among the six types of stacked graphenes; the maximum value is 0.357 eV. Both Z-Order and Z-AB stackings are good for the methane adsorption. As for the band gap, it would be smaller in armchair graphene, which means that the electronic performance of armchair graphene is better than zigzag graphene. The stacking does not cause big changes to band structure and DOS; it only makes some changes in the band gap. In the comparison of distance, both d1 and d2 of the Order stacked graphene are the largest among these three types of stacked graphenes. The distances of d1 and d2 in the AB-stacked graphene are the smallest. The distances of d1 and d2 in the ABC-stacked graphene are close to the AB-stacked graphene. The adsorption effect is the result of the interactions between graphene and methane combined with the change of graphene’s structure. The decrease of d2 can strengthen the adsorption effect of methane. Lastly, A-Order stacking processes the lowest formation energy in all types of stacked graphene. It is the best graphene structure for doping. In addition, it is also the best choice for methane adsorption. In summary, model 2 and Z-AB stacking are the best choices for the methane adsorption. When doping is needed to improve the methane adsorption effect, the A-Order stacking would be a good choice. These discoveries will contribute to the design and production of the graphene-based methane sensor.

## Figures and Tables

**Table 1:** The adsorption energy and the distances of zigzag graphene with Order stacking in the calculations of DFT and DFT-D, respectively.

**The Number of Layers (DFT)**	**Adsorption Energy (eV)**	**The Average Distance of Graphene (Å)**		**The Distance between Graphene and Methane (Å)**
	**-**	**d1**	**d2**	**d3**
1	50	-	-	3.658
2	62	4.147	3.929	4.192
3	99	4.151	4.133	4.149
4	138	4.306	4.077	3.737
**The Number of Layers (DFT-D)**	**Adsorption Energy (eV)**	**The Average Distance of Graphene (Å)**		**The Distance between Graphene and Methane (Å)**
	**-**	**d1**	**d2**	**d3**
1	255	-	-	3.337
2	277	3.422	3.420	3.354
3	308	3.418	3.415	3.352
4	351	3.416	3.415	3.360

**Table 2:** The adsorption energy, the distance, and the band gap of models 1, 2, and 3, respectively.

Model	Adsorption Energy		Distance		Band Gap			
	Zigzag (meV)	Armchair (meV)	Zigzag (Å)	Armchair (Å)	Zigzag		Armchair	
					Graphene (eV)	m-Graphene (eV)	Graphene (eV)	m-Graphene (eV)
1	242	217	3.363	3.362	0	0.006	0	0
2	255	230	3.337	3.336	0	0.007	0	0
3	243	218	3.352	3.355	0	0	0	0

**Table 3:** The average interlamellar distance of optimized graphene (d1) and methane-graphene (d2) and the distance between graphene and methane (d3).

**The Number of Layers in Order-Graphene**	**The Average Distance of Z-Graphene (Å)**		**The Distance between Z-Graphene and Methane (Å)**	**The Distance between A-Graphene and Methane (Å)**	**The Average Distance of A-Graphene (Å)**	
	**d1**	**d2**	**d3**	**d1**	**d2**	**d3**
1	-	-	3.337	-	-	3.336
2	3.422	3.420	3.354	3.450	3.454	3.355
3	3.418	3.415	3.352	3.435	3.434	3.350
4	3.416	3.415	3.360	3.435	3.434	3.370
**The Number of Layers in AB-Graphene**	**The Average Distance of Z-Graphene (Å)**		**The Distance between Z-Graphene and Methane (Å)**	**The Average Distance of A-Graphene (Å)**		**The Distance between A-Graphene and Methane (Å)**
	**d1**	**d2**	**d3**	**d1**	**d2**	**d3**
1	-	-	3.337	-	-	3.336
2	3.301	3.290	3.351	3.308	3.298	3.353
3	3.288	3.286	3.356	3.293	3.292	3.358
4	3.283	3.282	3.366	3.292	3.291	3.371
**The Number of Layers in ABC-Graphene**	**The Average Distance of Z-Graphene (Å)**		**The Distance between Z-Graphene and Methane (Å)**	**The Average Distance of A-Graphene (Å)**		**The Distance between A-Graphene and Methane (Å)**
	**d1**	**d2**	**d3**	**d1**	**d2**	**d3**
1	-	-	-	-	-	-
2	-	-	-	-	-	-
3	3.289	3.288	3.356	3.295	3.296	3.329
4	3.285	3.287	3.340	3.291	3.299	3.367
